# Soft-embalmed human breast tissue as a model for pre-clinical trials of HIFU - preliminary results

**DOI:** 10.1186/2050-5736-3-S1-P44

**Published:** 2015-06-30

**Authors:** Joyce Joy, Yang Yang, Ioannis Karakitsios, Roos Eisma, Colin Purdie, Andreas Melzer, Sandy Cochran, Sarah Vinnicombe

**Affiliations:** 1 University of Dundee, Dundee, United Kingdom

## Background/introduction

Around 52,000 people are diagnosed with breast cancer each year in the UK. With the controversy around over diagnosis arising from the breast screening programme there is intense interest in the possibility of safe effective non-invasive treatment of cancers. As a non-invasive method of lumpectomy, focused ultrasound surgery (FUS) may offers reduced risk of infection, fewer complications and a shorter recovery time. It also allows more precise treatment as a result of real-time guidance by magnetic resonance (MR) or ultrasound. Even though specially designed FUS transducers for breast cancer treatment are now becoming available, transducer efficacy needs to be tested with a suitable preclinical model. A specific issue is the accuracy of temperature monitoring of FUS with MRI in the breast, since the presence of large amounts of surrounding fat can impair temperature measurement with the proton resonant frequency. An appropriate anatomical model that enforces comparable physical constraints to the breast and that responds to FUS in the same way would be extremely advantageous. The aim of this feasibility study is to explore the use of soft embalmed cadaveric breast tissue for these purposes. We report here the early results of MRI-guided FUS experiments sonicating dissected breast samples from a soft-embalmed human cadaver with a high body mass index (BMI).

## Methods

A specially developed MRI compatible chamber and sample holder was developed to secure the sample and ensure reproducible sonications at the transducer focus. A HIFU transducer of frequency of 1.09 MHz and focal length of 69mm was used for sonications. An MRI compatible thermocouple was used to measure the temperature rise induced in the chosen tissues by sonications. The efficacy of sonication was first studied with chicken breast and porcine tissue. The experiments were then repeated with the dissected fatty breast tissue samples from soft-embalmed human cadavers.

## Results and conclusions

**Fig 1:**
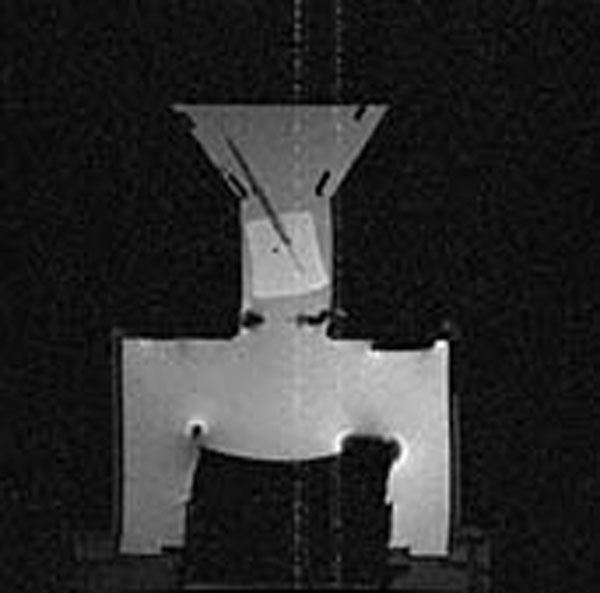
MRI Image of the chamber with the tissue showing the thermocouple tip at the ultrasound focus

After successful testing of the HIFU chamber with fresh animal tissue sonications, the experiments were repeated using cadaveric breast tissue. The thermocouple was placed at the focal zone of the FUS transducer and the position was confirmed using MR imaging (Fig 1). Lower temperature peaks & the absence of a visible lesion in the soft-embalmed breast tissue were investigated further with sonications of embalmed porcine and chicken breast tissue. Histopathological analysis of all the sonicated embalmed samples confirmed the absence of any discrete lesion. As with the embalmed chicken and porcine samples, observed temperature rises were lower in the embalmed human breast samples. Melting of fat during sonications resulted in movement of the thermocouple, resulting in irregular temperature measurements. The preliminary results of sonicating soft-embalmed human breast tissue are discouraging, with difficulty in creating visible lesions and irregular temperature rises due to the melting of fat. The latter reduces the tissue volume thus moving the tissue along with the thermocouple away from the ultrasound focus. The design of the chamber has now been modified to prevent the movement of the tissue during sonications. The new chamber will be tested in the near future and the results will be compared with the fresh human breast samples from mastectomy specimens. Results to date have confirmed the inability to produce a discrete lesion in soft embalmed tissue samples suggesting the need for fresh breast tissue samples for pre-clinical HIFU trails. However, more experiments are needed for firm conclusions.
